# *In-vitro *Study of Multifunctional PLGA-SPION Nanoparticles Loaded with Gemcitabine as Radiosensitizer Used in Radiotherapy

**DOI:** 10.22037/ijpr.2019.14050.12131

**Published:** 2019

**Authors:** Nima Hamzian, Maryam Hashemi, Mahdi Ghorbani, Seyed Amir Aledavood, Mohammad Ramezani, Mohammad Hossein Bahreyni Toosi

**Affiliations:** a *Department of Medical Physics, Faculty of Medicine, Shahid Sadoughi University of Medical Sciences, Yazd, Iran.*; b *Nanotechnology Research Center, Institute of Pharmaceutical Technology, Mashhad University of Medical Sciences, Mashhad, Iran. *; c *Department of Biomedical Engineering and Medical Physics, Faculty of Medicine, Shahid Beheshti University of Medical Sciences, Tehran, Iran.*; d *Cancer Research Center, Mashhad University of Medical Sciences, Mashhad, Iran. *; e *Pharmaceutical Research Center, Institute of Pharmaceutical Technology, Mashhad University of Medical Sciences, Mashhad, Iran. *; f *Department of Pharmaceutical Biotechnology, School of Pharmacy, Mashhad University of Medical Sciences, Mashhad, Iran. *; g *Medical Physics Research Center, Faculty of Medicine, Mashhad University of Medical Sciences, Mashhad, Iran.*

**Keywords:** Multifunctional drug delivery system, SPION-PLGA, Gemcitabine, Radiotherapy, Radiosensitizer

## Abstract

This study aimed to modify the biological response of cells to ionizing radiation by combination therapy using radio-sensitizer agent and anticancer drug. Super paramagnetic iron oxide nanoparticles (SPIONs) were prepared and used with gemcitabine (Gem). These two agents were encapsulated simultaneously intopoly (D, L-lactic-co-glycolic acid) (PLGA) to form multifunctional drug delivery system. The physicochemical characteristics of the nanoparticles (NPs) were studied. The dose enhancement ratio (DER) of various treatment groups was calculated and compared using human breast cancer cell line (MCF-7). The DER for PLGA-SPION-Gem was the highest at 1 Gy^60^Co (3.18). Cumulative effect from simultaneous use of two radiosensitizer (Gem and SPIONs) was observed. Thus, we have successfully developed PLGA NPs loaded with gemcitabine and SPIONas a radiosensitizersystem which potentially could be used in radiotherapy.

## Introduction

To increase the treatment efficiency of radiation therapy, many efforts have been focused on the enhancement of the dose delivery or modification of the biological response to ionizing radiation (1). Additionally, collective or synergistic effects against a tumor without considerable increase in toxicity of normal tissues, can lead to therapeutic benefits (1). For example, combination of radiotherapy and chemotherapy improves the survival of the patients bearing resistant tumors to ionizing radiation.

A large variety of drugs have produced moderating effect of response to radiation. Among the available drugs, the compounds that cause selective sensitization of hypoxic tumor cells are most important. These compounds are known as radiosensitizers (2, 3). In combination with radiotherapy, gemcitabine (2′, 2′-difluoro-2′-deoxycytidine) (Gem) an effective drug for the treatment of various solid tumors and a number of hematologic malignancies (4-7) was also considered as a radiosensitizer (1). *In-vitro* studies showed that gemcitabine at non-toxic concentrations in combination with ionizing radiation had an enhanced cytotoxic activity in colorectal, breast, ovarian, and lung cancers and delayed tumor growth in animal models (8-11). However, there is not enough data to confirm the mechanism of radiosensitization by gemcitabine.

Gemcitabine has chemical instability, poor cellular uptake and a very short half-life in plasma requiring higher doses which may result in systemic toxicity. To overcome these major disadvantages, magnetic nanoparticles (MNPs) have been investigated as carriers for targeted drug delivery (12-14). These nanoparticles are considered because of their high magnetic responsibility, biodegradability, biocompatibility, high delivery efficiency, and potential targeting ability (15-17). 

Moreover, superparamagnetic iron oxide nanoparticles (SPIONs) are approved for clinical use by the US Food and Drug Administration (FDA) (18).

In a number of studies, SPIONs have been used for diagnostic and therapeutic applications (19-22). For this purpose, magnetic Fe_3_O_4_ nanoparticles were first prepared and then encapsulated in the polymeric carriers (13, 14, 23 and 24).

As a radiosensitizer, SPION may increase the efficiency of radiotherapy through generation of photoelectrons and Auger electrons due to the interaction with X-rays and gamma rays and induction of reactive oxygen products (ROS) and therefore leads to oxidative stress (21).

Recently, the use of biodegradable copolymers of poly lactic-co-glycolic acid (PLGA) for the delivery of chemotherapeutic drugs has enhanced the treatment efficiency of tumors in a wide range of cancer types (24-29). PLGA has been approved by the FDA for human use (25). The advantages of this polymer are biodegradability, biocompatibility, ease of accumulation within the tumor, rapid clearance from biological systems, and high efficiency of drug delivery (30, 31).

This study was aimed to increase the efficiency of radiotherapy with combination of two radiosensitizers. Thus, gemcitabine and SPIONs are co-encapsulated in PLGA (as PLGA-SPION-Gem nanoparticles) simultaneously to take the advantages of synergistic effects of nanoplatform with lower toxicity. The physicochemical properties of nanoparticles and their radiosensitization potentials were evaluated in human breast cancer cell line (MCF-7). 

## Experimental

*Materials*

Ferric chloride hexahydrate (FeCl_3_⋅6H_2_O, 98%), ferrous chloride tetrahydrate (FeCl_2_⋅4H_2_O), and ammonium hydroxide (25% w/v) were purchased from Fluka (Buchs, Switzerland). Poly (D, L-lactic-co-glycolic acid) (PLGA) (average Mw: 17,000-35,000; lactic acid: glycolic acid = 50:50) and 3-(4,5-dimethylthiazol-2-yl)-2,5-diphenyltetrazolium bromide (MTT) were obtained from Sigma-Aldrich (Munich, Germany). Gemcitabine hydrochloride was purchased from Eurasia Co., Ltd. (Delhi, India). Roswell Park Memorial Institute (RPMI) 1640 medium, fetal bovine serum (FBS) and trypsin were purchased from GIBCO (Gaithersburg, Germany). Polyvinyl alcohol (PVA, 87-89% hydrolyzed, average MW = 88,000-97,000) and other solvent and chemical reagents were procured from Merck (Germany) without further purification.

*Synthesis of SPIONs*

SPIONs were synthesized using a co-precipitation method as described in our previous study (24). Briefly, after removing of O_2_ from deionized water, FeCl_2_.4H_2_O and FeCl_3_.6H_2_O were added to the vigorously stirred water followed by quickly addition of oleic acid to the mixture placed in a water bath (75-80 °C). SPIONs were formed by adding of NH_4_OH to the previous reaction solution. The product was washed three times with deionized water, separated using a permanent magnet and lyophilized.

*Preparation of PLGA-SPION-Gem nanoparticles*

According to our previous research, double emulsion method (W_1_/O/W_2_) was used for the preparation of PLGA-SPION-Gem NPs (24). Briefly, gemcitabine solution was added to organic phase containing SPION and PLGA in chloroform and dichloromethane, respectively and emulsiﬁed by probe sonication (Fisons Instruments Ltd., Crawley, UK) for 1 min (0.6 Hz frequency, 90 amplitude) (W_1_/O). The primary water-in-oil (W/O) emulsion was added drop wise to PVA solution (5%, w/v) and emulsified for 10 min using a probe sonicator (W_1_/O/W_2_). To evaporate the organic solvent, the resulted solution was diluted in 10 mL aqueous PVA solution (0.1%, w/v) under stirring at room temperature overnight. Then, the nanoparticles were collected by centrifugation at 14000 rpm for 25 min and washed three times with deionized water. Finally, the products were freeze-dried.

The PLGA-Gem, PLGA-SPION, and PLGANPs were prepared using similar method as described in previous study.

*Nanoparticles characterization*

Loading content and encapsulation efﬁciency of gemcitabine and SPIONs was obtained using a UV-Vis spectrophotometer (*λ *= 268 nm) (Shimatzu, Tokyo, Japan) and atomic absorption spectrophotometer (CTA-3000, ChemTech, UK), respectively and calculated using the following Equations: 

Loading contents (%) = (Drug weight in the nanoparticles/Weight of nanoparticles) × 100 

Encapsulation Efficiency (%) = (Residual drug in the nanoparticle/Initial feeding amount of drug) × 100

Hydrodynamic diameter, poly disparity index (PDI), and zeta potential of nanoparticles were determined with dynamic light scattering method with zetasizer (NANO-ZS, Malvern, UK).

The particle size and morphology of the magnetic nanoparticles were observed by transmission electron microscopy (LEO 910, Zeiss, Germany-300 mesh) and the surface morphology of PLGA-SPION-Gem nanoparticles was observed by Atomic Force Microscopy (AFM, model: Nano Wizard II NanoScience AFM, JPK Instruments Inc., Germany).

*Cell culture and Non-toxic concentration evaluation*

MTT assay was used to evaluate the toxicity of gemcitabine and prepared formulations. To determine the IC_10_ for all PLGA, PLGA-SPION, PLGA-Gem, PLGA-SPION-Gem, and gemcitabine alone, the MCF-7 cells were seeded into 96-well plates at density of 1 × 10^4^ cells/well. After 24 h of incubation, different concentrations of gemcitabine (1 to 500 nM) and its equivalent concentrations of other formulations were dispersed in RPMI containing 10% FBS and were replaced with equal volume of RPMI in each well. After 48h of incubation, MTT assay was used and the results were obtained using an ELISA microplate reader (TECAN inﬁnite M200, Switzerland) at 570/630 nm wavelength. 

*Radiation treatment of cells containing nanoparticles*

To study the effect of radiation treatment, the cells were seeded into 96-well plates at density of 2 × 10^3^ cells/well and incubated for 24 h. Then, the medium was replaced with equivalent concentration of 10 nM gemcitabine (IC_10_) of various treatment groups in RPMI for 24 h. Then, the cells were irradiated with different doses of ^60^Co radiation (0.5, 1, 3, 5 and 7 Gy) with a dose rate of 0.87 cGy/min. After 7 days of incubation, the cell viability was determined by MTT assay as described above. Different groups of the cells without radiation exposure were considered as the control groups. Dose enhancement ratio (DER) was calculated by dividing the surviving fraction of the cells without drug treatment to the surviving fraction of the cells with drug treatment for each dose of radiation.

*Statistical analysis*

For statistical analysis, SPSS 16.0 software was used. The students′ *t*-test was used for comparison of individual groups and one-way ANOVA was used to obtain statistical differences of multiple groups. Probabilities of *p *< 0.05 were considered as signiﬁcant. The results were reported as mean ± standard deviation (SD).

## Results and Discussion

Radiotherapy is one of the most common treatment modalities for cancer. Either improvement of the radiation dose delivery system or modification of the biological response to ionizing radiation has led to the efficiency enhancement of radiation therapy to kill tumor cells with no or negligible side effects on surrounding healthy tissues (1). The current study aimed to modify the biological response to ionizing radiation by using combined radiosensitizers in one polymeric delivery system. 

Thus, superparamagnetic iron oxide nanoparticles were synthesized and co-encapsulated into PLGA NPs with gemcitabine as a chemotherapeutic and radiosensitizers agent. The physicochemical characteristics of the polymeric NPs were studied and the effect of the new formulation on the efficiency of radiotherapy was evaluated in MCF-7 cell line.

*Physicochemical properties of synthetized NPs*

**Figure 1:**
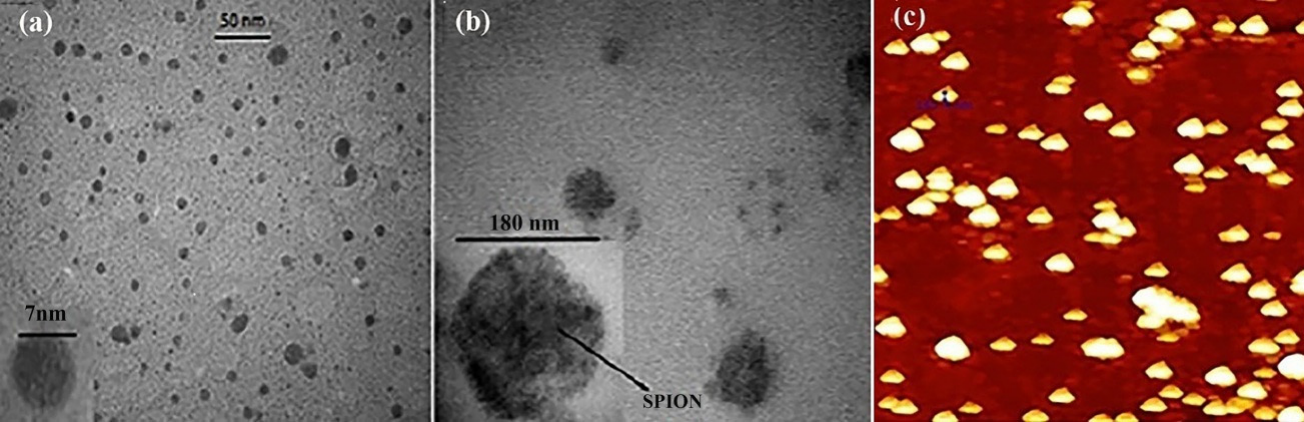
TEM and AFMimages of nanoparticles. (a) TEM image of SPIO nanoparticles, (b) TEM image of PLGA-SPION-Gem nanoparticles, (c) AFM image of PLGA-SPION-Gem nanoparticles (24).

Figure 1 illustrated the morphology of nanoparticles obtained by TEM and AFM. As shown in Table 1, the SPION encapsulation efficiency and loading content in polymeric nanoparticles are about 50% and 28%, respectively. Gemcitabine loading capacity in polymeric nanoparticles is 2.8% and 3.8% for the PLGA-SPION-Gem and PLGA-Gem formulations, respectively. Efficiency of gemcitabine encapsulation is 16.1 ± 2.2 (%) and 13.2 ± 1.3 (%) in these formulations, respectively.

Higher SPION encapsulation (4 folds) in PLGA-SPION-Gem formulation compared to gemcitabine may be effective for radiosensitivity and imaging purposes. As SPIONs are not toxic, they are preferred to be used at higher encapsulation efficiency in polymeric nanoparticles as radiosensitizer whereas gemcitabine with high cytotoxicity may produce sever side effect at higher doses and therefore, co-encapsulation of SPION at higher concentration and gemcitabine at lower concentration in PLGA could be an strategy to minimize the toxicity of the formulation and maximize the treatment efficiency of formulation.

**Figure 2:**
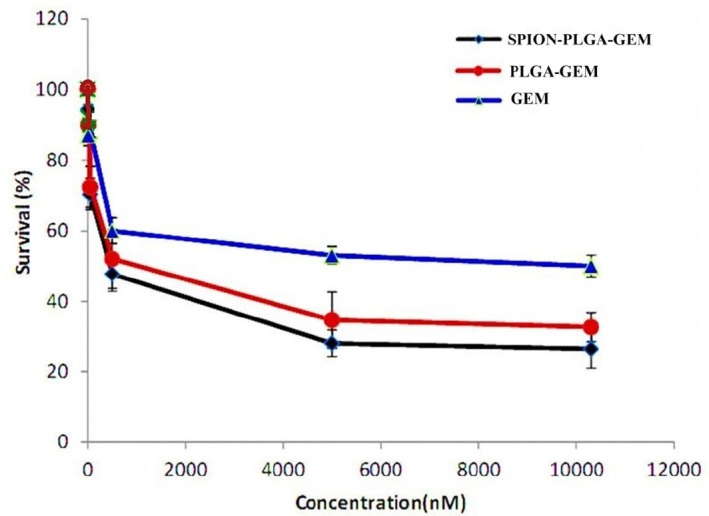
Viability (%) of MCF-7 cells of various treatment groups after 48 h incubation. The concentrations of formulations are equivalent to the concentration of 0.01 µM to 10.3 µM of gemcitabine (24).

**Figure 3.**
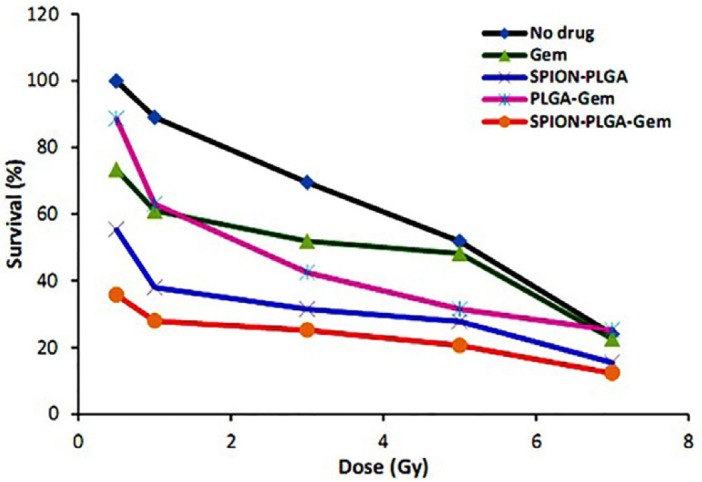
Viablity (%) of MCF-7 cells in various treatment groups after 7 days of incubation. Concentration used for all formulations contained 10 nM gemcitabine (IC_10_). The cells were exposed to serial doses of gamma radiation of ^60^Co (0.5, 1, 3, 5 and 7 Gy) with dose rate of 0.87 cGy/min

**Figure 4:**
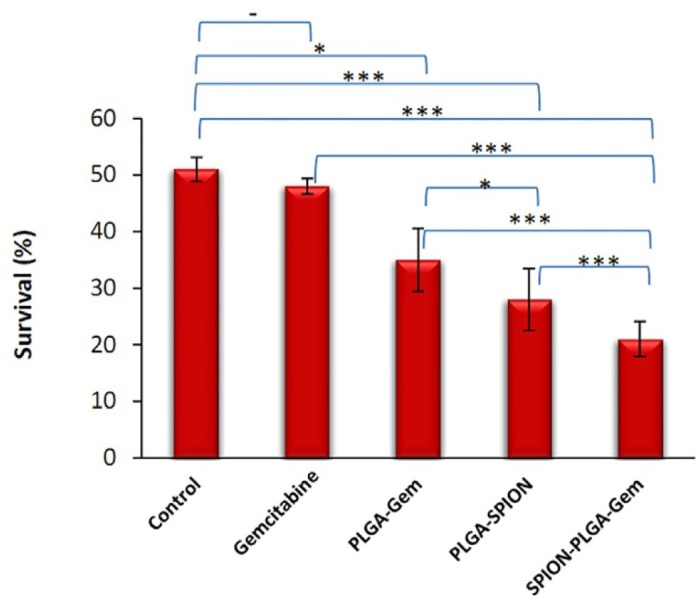
Viability (%) of MCF-7 cells of various treatment groups after 7 days incubation. Concentration used for all formulations contained 10 nM gemcitabine (IC_10_). The cells were exposed to 5 Gy (D_50_ of control group) ^60^Co gamma radiation with dose rate of 0.87 cGy/min. ^*^P ≤ 0.05, ^**^P ≤ 0.01, ^***^P ≤ 0.001

**Table 1:** Loading content and encapsulation efficiency of gemcitabine and (or) SPION. Encapsulation efﬁciency of SPION and gemcitabine were determined in triplicate using atomic absorption spectrophotometer and UV-Vis spectrophotometer, respectively

**Encapsulation efficiency (%)**			**Loading content (%)**	
**Formulation**				
	**Gem**	**SPION**	**Gem**	**SPION**
PLGA-SPION	-	50.1 ± 1.1	-	28.6
PLGA-Gem	13.2 ± 1.3	-	3. 8	-
PLGA-SPION-Gem	16.1 ± 2.2	48.2 ± 2.1	2.8	27.8

**Table 2:** Size, zeta potential and PDI of nanoparticles

**Formulation**		**Z ** **ave ** **(nm)** ** **(mean ± SD)**	**PDI** * **(mean ± SD)**	**Zeta potential (mV) (mean ± SD)**
SPION	2.2 ± 20.1		0.20 ± 0.05	+20.2 ± 2.0
PLGA	190.6 ± 8.6		0.03 ± 0.01	-15.3 ± 0.5
PLGA-SPION	170.3 ± 4.6		0.16 ± 0.04	-12.1 ± 1.0
PLGA-Gem	175.2 ± 8.3		0.05 ± 0.02	-14.2 ± 1.1
PLGA-SPION-Gem	180.2 ± 10.3		0.08 ± 0.02	-13.3 ± 1.0

*Polydispersity index (PDI), size and zeta potential (mV) were obtained by DLS.

**PLGA-SPION-Drug (without Gem) formulations were previously reported with approximately similar synthesis and preparation methods.

**Table 3:** Viablity (%) of various treatment groups containing gemcitabine (IC_10_ concentration) exposed to 5 Gy (D50 of control group) ^60^Co gamma radiation. Dose enhancement ratios were calculated at 1 and 5 Gy doses

**Formulation**	**Viability**	***P*** **-Value** **†**		**DER**		
	**5Gy (** **60** **Co) IC Gem** *	**1Gy**	**5Gy**	**1Gy**		**5Gy**
Control	50.0 100.0	-	-			
Gem	48.4 90.1	0.011	0.189	1.46		1.03
PLGA-Gem	35.1 89.2	0.001	0.014	1.63		1.42
PLGA-SPION	28.2 97.1	0.000	0.001	2.34		1.77
PLGA-SPION-Gem	20.1 88.3	0.000	0.000	3.18		2.49

^*^Without radiation, ^†^Compared with the control group.

As shown in Table 2, the average hydrodynamic diameter of SPIONs was 20.0 ± 2.0 nm and the size of SPIONs obtained by TEM is 7.0 ± 0.5 nm. The average hydrodynamic diameter of polymeric nanoparticles with minor differences was less than 200 nm. 

There is a consistency between the sizes of NPs prepared in this study with those reported previously using similar preparation methods (24).

*Cytotoxicity assay*

With regard to the drug release profile of the NPs in the previous study, it could be concluded that the toxicity assay of the nanoparticles should be performed when the cells were incubated with NPs for 48 h. However, considerable difference was not observed when the cells where incubated for either 48 or 72 h (24). 

Non-toxic concentration of gemcitabine was considered as IC_10_ which was 10 nM for MCF7 cells incubated for 48 h which was in accordance with the results reported elsewhere (Figure 2) (1). The toxicity of the other formulations containing equal concentration of gemcitabine (*i.e.* 10 nM) had no considerable difference with gemcitabine alone showing similar IC_10_. PLGA-SPION formulation at much higher concentrations (equivalent to 25 μM of gemcitabine in other formulations) showed no considerable toxicity (less than 10% toxicity). This low toxicity suggested that loading of higher concentration of SPIONs in different formulations has a great advantage for either therapeutic or diagnostic applications.

*Simultaneous cell treatment with irradiation and PLGA-based nanoparticles*

Viability (%) of MCF-7 cells in various treatment groups after 7 days of incubation are illustrated in Figure 3 and statistical differences between various groups at 5 Gy are also illustrated in Figure 4. The DER of gemcitabine, PLGA-Gem, PLGA-SPION, and PLGA-SPION-Gem nanoparticles at 5 Gy (D50 of MCF-7 cells without drug) and 1.0 Gy irradiation are illustrated in Table 3.

In the current study, the DER of 10 nM gemcitabine in 1 Gy^60^Co gamma irradiation has the maximum value of 1.46 (*P *≤ 0.01 compared with the control group). This value decreased with increasing the radiation dose approaching 1.0 for 5 Gy (equivalent to D50 for the control group), and reached 1.0 for 7 Gy (Figure 3).

This suggested that at higher radiation doses, radiosensitization of gemcitabine was not considerably high with no significant difference with the control group (*P *> 0.05). This result seems to be reasonable and could well be attributed to the fact that by increasing the radiation dose, the cell damage which was directly caused by radiation surpassed the damage exerted by the radiosensitizer in such a way that the sensitizing effect of gemcitabine was less pronounced at higher doses.

Depending on the cell type used, concentration of gemcitabine, and the schedule of administration, the effect of irradiation was varied. *In-vitro* studies on radiosensitivity of gemcitabine have reported DERs between 1.1 to 3.0. The concentration of gemcitabine used in these studies was mostly between 0 and 10 nM. In most studies, concentration equivalent to IC_10_ of gemcitabine (10 nM) has been used to evaluate the radiosensitivity activity (1). This is also in accordance with the results of recent studies (32-34). While the gemcitabine dose as radiosesitizer varies in different cell lines, in the study by Robinson and Shewach, MCF7 cells were exposed by a concentration of 10 nM gemcitabine and were irradiated at 0-10 Gy immediately after incubation and the dose enhancement ration of 1.63 has been reported (35).

In the case of PLGA-Gem nanoparticles, DER with 5 and 1 Gy was 1.42 and 1.63, respectively showing more radiosensitivity activity compared to gemcitabine alone (*P *≤ 0.05 compared with the control group at both doses).

As it can be seen from the data presented in Table 3, at irradiation dose of 5 (D50 of the control group) and 1 Gy, the DER of PLGA-SPION nanoparticles were 1.78 and 2.34, respectively. It clearly indicated that the radiosensitization activity of PLGA-SPION nanoparticles is more than PLGA-Gem nanoparticles (*P *≤ 0.05 at 5 Gy). This is may be partly due to the fact that the concentration of SPION encapsulated in the formulation of PLGA-SPION is almost 33 folds more than the concentration of gemcitabine loaded in the formulation of PLGA-Gem. In fact, low toxicity of SPION in biological environments provides the possibility of using higher concentrations which is a great advantage for both diagnostic and therapeutic applications.

In the PLGA-SPION-Gem formulation, different encapsulated efficiency of gemcitabine and SPION in PLGA would allow to use gemcitabine at concentration with no obvious toxicity (IC_10_) while the radiosensitization activity of the formulation could be optimized using high concentrations of SPION. This would also overcome the need for increasing the loading efficiency of gemcitabine in this formulation. As shown in Figure 3, the radiosensitization activity of polymeric nanoparticles was significantly higher than that for gemcitabine alone. PLGA-SPION-Gem nanoparticles exhibited the highest radiosensitization activity compared with the control group (*P *≤ 0.001). According to the data presented in Table 3, the DER of PLGA-SPION-Gem nanoparticles in 5 (D50 of the control group) and 1 Gy were 2.49 and 3.18, respectively.

According to Figure 4, there is no significant difference between the radiosensitization activity of PLGA-Gem and Gem alone compared to the control group (*P *= 0.189 and *P *= 0.014, respectively), whereas for PLGA-SPION and PLGA-SPION-Gem nanoparticles, the difference was significant (*P *≤ 0.001). However, as mentioned for 1 Gy dose, the radiosensitization activity of PLGA-Gem and Gem were also significant compared to the control (*P *≤ 0.01 and *P *≤ 0.05, respectively). 

As indicated in Figure 4 and Table 3, radiosensitization activity of PLGA-SPION-Gem nanoparticles was significantly higher than that of PLGA-SPION and PLGA-Gem nanoparticles (with DER of 2.49 versus 1.77 and 1.42, with *P *≤ 0.001 for both). The enhancement percentage of DER for PLGA-SPION-Gem (149%) was clearly more than the sum of the enhancement percentages of DER for both PLGA-SPION and PLGA-Gem (77% and 42%, respectively) which is an indication of a synergistic effect of combined encapsulation of gemcitabine and SPION in PLGA-SPION-Gem formulation. This phenomenon was more apparent when the comparison was made in 1 Gy irradiation dose where control group with no drug content, showed a viability of about 90% (*i.e.* 218% >134% + 63%). 

Although there may be no obvious explanation for dose enhancement effect of PLGA-SPION-Gem formulation compared to two other polymeric formulations, several reasons could be mentioned as follows: a) An increase in the efficiency of the production of H˚ and OH˚ free radicals caused by simultaneous presence of gemcitabine and iron ions; b) production of a synergistic effect due to the consolidation of damage caused by free radicals produced by the interaction of radiation with gemcitabine molecules due to the existence of oxygen in SPIONs; c) production of a synergistic effect resulted from the increased efficiency in the production of H˚ and OH˚ free radicals from interaction of secondary radiations with gemcitabine molecules. These secondary radiations are generated by the photoelectric interaction between the primary radiations with iron atoms. However, as shown in Figure 4, at higher doses (7 Gy and higher), the difference in radiosensitivity activity between groups decreased and became statistically insignificant. The highest radiosensitization activity of all formulations was observed at low dose of 1 Gy whereas the sensitization effect was less evident at higher doses. The reason for this effect could be attributed to the fact that by increasing the irradiation dose, the damages were directly caused by the radiation, surpassing those caused by radiosensitization activity of gemcitabine and SPIONs encapsulated in PLGA. Therefore, it is expected that PLGA-SPION-Gem radiosensitization activity plays a better role in the fractionated treatment that is common in radiotherapy.

## Conclusion

We have successfully developed a PLGA-based formulation containing gemcitabine and SPION as a system for combined delivery of two radiosensitizers which could be used for radiotherapy purposes. According to the results of this study, PLGA-SPION-Gem nanoparticles had an excellent radiosensitization activity and simultaneous use of SPION and gemcitabine could lead to the enhancement of efficiency of fractionated radiation therapy. Furthermore, this formulation improves the instability of gemcitabine and therefore is an appropriate system for delivery of gemcitabine.

Higher loading efficiency of SPION (4 folds) in PLGA-SPION-Gem formulation compared to gemcitabine could provide an effective system for therapeutic and imaging purposes. 
